# Revisiting the hyperdominance of Neotropical tree species under a taxonomic, functional and evolutionary perspective

**DOI:** 10.1038/s41598-021-88417-y

**Published:** 2021-05-05

**Authors:** Gabriel Damasco, Christopher Baraloto, Alberto Vicentini, Douglas C. Daly, Bruce G. Baldwin, Paul V. A. Fine

**Affiliations:** 1 Department of Integrative Biology, University of California, Berkeley, CA 94720-3140 USA; 2 Departamento de Botânica e Zoologia, Universidade Federal do Rio Grande do Norte, Natal, RN 59072-970 Brazil; 3 Institute of Environment, Florida International University, Miami, FL 33133 USA; 4 Programa de Pós-Graduação em Botânica, Instituto Nacional de Pesquisas da Amazônia, Manaus, AM 69080-971 Brazil; 5 Institute of Systematic Botany, The New York Botanical Garden, Bronx, NY 10458 USA

**Keywords:** Phylogenetics, Population genetics, Biodiversity, Biogeography, Ecophysiology

## Abstract

Recent studies have leveraged large datasets from plot-inventory networks to report a phenomenon of hyperdominance in Amazonian tree communities, concluding that few species are common and many are rare. However, taxonomic hypotheses may not be consistent across these large plot networks, potentially masking cryptic diversity and threatened rare taxa. In the current study, we have reviewed one of the most abundant putatively hyperdominant taxa, *Protium heptaphyllum* (Aubl.) Marchand (Burseraceae), long considered to be a taxonomically difficult species complex. Using morphological, genomic, and functional data, we present evidence that *P. heptaphyllum *sensu lato may represent eight separately evolving lineages, each warranting species status. Most of these lineages are geographically restricted, and few if any of them could be considered hyperdominant on their own. In addition, functional trait data are consistent with the hypothesis that trees from each lineage are adapted to distinct soil and climate conditions. Moreover, some of the newly discovered species are rare, with habitats currently experiencing rapid deforestation. We highlight an urgent need to improve sampling and methods for species discovery in order to avoid oversimplified assumptions regarding diversity and rarity in the tropics and the implications for ecosystem functioning and conservation.

## Introduction

Accurately defining species and their distributions is critical to understanding the processes responsible for both the generation and the maintenance of biodiversity. The increasing availability and analysis of species distribution data through forest inventory networks (e.g., GBIF, ATDN, RAINFOR, PPBio, DRYFLOR, 2ndFOR) has resulted in important conclusions about species diversity (e.g.,^1–3^) and related ecosystem processes (e.g.,^4–6^). Yet, despite the scientific advances made possible by the analysis of large dataset networks, the diversity of tropical species remains critically understudied, even for well-studied groups such as Amazonian trees^7,8^.

Estimates regarding the total known species richness of the Amazonian tree flora have been recently debated. The numbers vary from 6727^9^ (based principally on records from regional floras) to 10,071 species^10^ (based on plot-based checklists screened for taxonomic validity), but the actual richness is expected to reach over 16,000 species^11^, given the large number of unsampled areas and the high diversity of extremely rare trees^1^. Discrepancies between empirical and estimated values are usually attributed to at least one of three explanations: (i) undersampled regions; (ii) undescribed or undetermined taxa, collected but yet to be identified; and (iii) the presence of species complexes or cryptic lineages for which subtle morphological distinctions may have been recognized but no genetic data have been included to establish whether putative taxa are distinct. Although 1068 new species have been added since ter Steege and collaborators published the first ATDN (Amazon Tree Diversity Network) checklist^10^, there have been no systematic efforts to review the taxonomy of hyperdominant taxa (defined as the 227 most common species accounting for more than half of all trees in the Amazon Basin^1^), especially those considered taxonomically difficult groups, morphospecies, or species complexes.

Potential species complexes are characterized by at least one of the following criteria (reviewed in ^12^): (i) morphologically cryptic variation, (ii) recent evolutionary divergence and genetic introgression, (iii) broad geographic range with incomplete sampling, and (iv) taxonomic redundancy (i.e., numerous synonyms). Plant species complexes are fairly common in the Amazon region (e.g.,^13–15^) and based on the criteria above, we speculate that at least half of the 50 most hyperdominant Amazonian trees^1^ may represent species complexes. If some or all of these hyperdominant species are actually species complexes that warrant treatment as multiple species or separately evolving taxa, numerous important consequences would arise. For example, resolution of such complexes would refine predictions about Amazonian species diversity and identify previously unknown and potentially threatened taxa and key habitats as new conservation priorities. In addition, it would enhance the understanding of ecosystem processes and global change if newly described taxa exhibit differential functional responses to variation in abiotic and/or biotic conditions.

Here, we review a putatively dominant and widespread plant species, *Protium heptaphyllum*, by conducting extensive population-level sampling across its geographic range. As circumscribed to date, *P. heptaphyllum* was listed as the 12th most common tree species in Amazonia^1^, occurring also in the Cerrado and Atlantic forest of Brazil. *Protium* is one of the taxonomically best- studied genera in the Neotropics (e.g.,^16–19^) and it was recently listed the second most abundant tree genus in the Amazon Basin^1^. We combined morphological, ddRADseq (double digest Restriction site Associate DNA sequencing), and physiological data to answer the following questions: (i) Does this hyperdominant tree represent a single taxon or multiple taxa? (ii) What processes appear to have generated and maintained evolutionary divergence of lineages within *P. heptaphyllum*? (iii) To what extent do functional strategies vary across lineages? The answers to these questions are discussed here in detail as they play an important role in understanding the diversity, ecosystem processes and conservation of Amazonian forests.

## Results

### Morphological and molecular species delimitation

**Figure 1:**
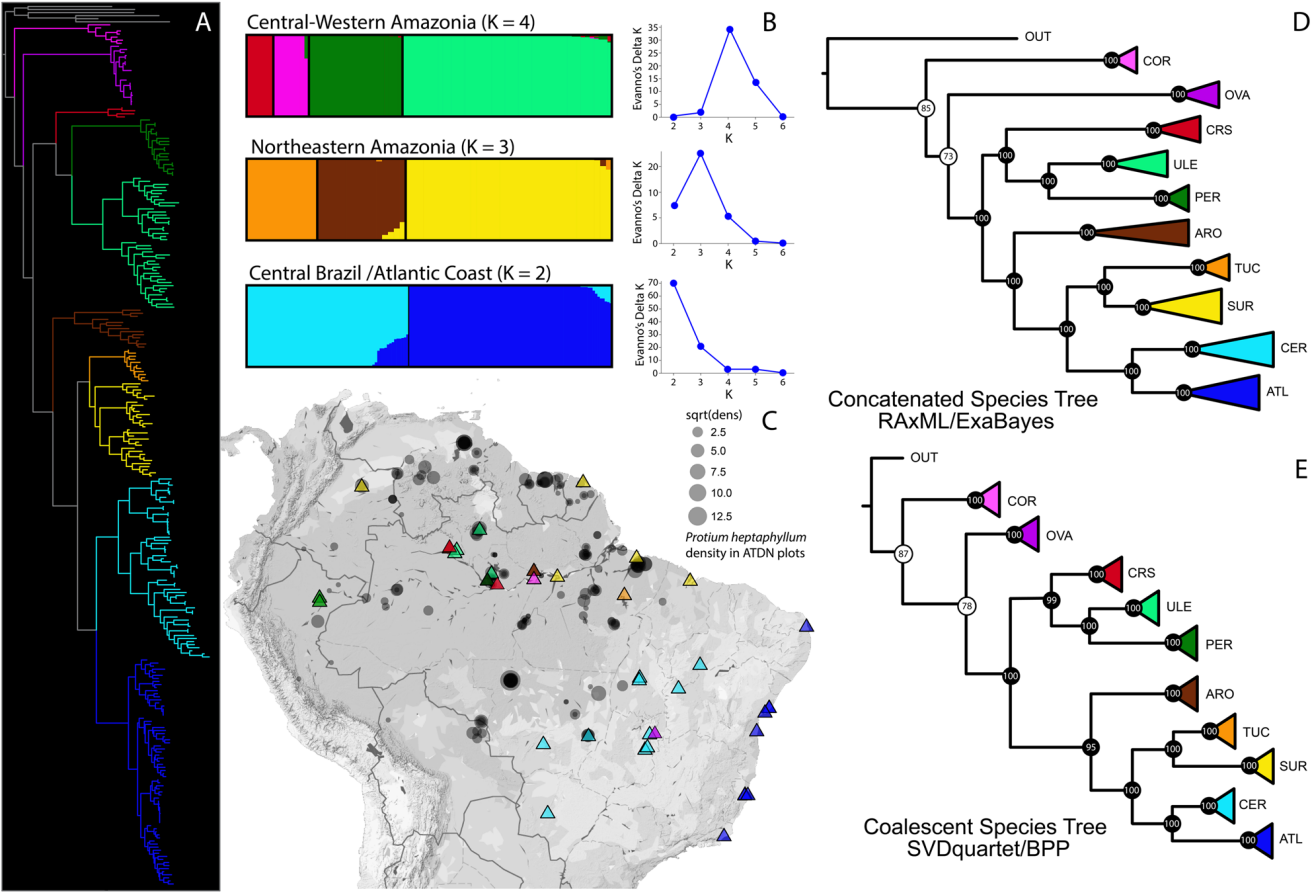
Phylogenetic reconstruction and population genetic structure of *Protium heptaphyllum s.l.* (Aubl.) Marchand. **(A)** Maximum likelihood (RAxML) phylogeny of *P. heptaphyllum* inferred with 1000 bootstrap replicates using the GTRGAMMA substitution model. The dataset consists of 285 samples and 6,234 loci. Trees were inferred from concatenated data sets using RAxML version 8.2.1060 (https://github.com/stamatak/standard-RAxML) and consensus tree visualized using the R package ape version 5.3 (http://ape-package.ird.fr/). **(B)** STRUCTURE analysis identified three main clusters that encompasses the Central/West and North/East of Amazonia, and the Central and Atlantic Coast of Brazil. The bar graphs represent a subsequent STRUCTURE run within each geographic group based on a combination of 10 replicate runs. Colors indicate the assignment of each individual to a particular genetically similar cluster. The number of clusters within each geographic region were identified based on the ΔK statistics. Analysis and figure visualization were performed in STRUCTURE version 2.3.4 (http://web.stanford.edu/group/pritchardlab/structure.html). **(C)** The location of each sampled population is displayed on the map (triangle symbols). Colors indicate the population assignment to a particular genetic cluster. Circle symbols corresponds to the plot location where *P. heptaphyllum s.l*. was sampled by the Amazon Tree Diversity Network (ATDN). Circle sizes are equivalent to the square root of density values per hectare in each of the ATDN plots. Figure generated using the R package ggmap version 3.0.0 (https://www.rdocumentation.org/packages/ggmap/versions/3.0.0). **(D,E)** Species tree inference based on concatenated data matrices ran in RAxML and ExaBayes software and based on the multispecies coalescent model ran in SVDquartet and BPP software, respectively. Node values represent bootstrap support and posterior probabilities for each clade. White circles correspond to support values that are not identical between RAxML bootstrapping and ExaBayes posterior probabilities. In this case, the lower support value was displayed in the figure. Both species trees were visualized using FigTree version 1.1 (http://tree.bio.ed.ac.uk/software/figtree/). See Figure S2 and Table S2 for more information about the putative new taxa.

We find that *Protium heptaphyllum s.l.* represents at least eight different taxa based on both morphological and molecular evidence (Fig. 1, Figure S2, Table S2). The STRUCTURE analysis of genomic data recognized three major clusters that represent a broad pattern of genetic differentiation among major biogeographic regions in South America: (i) Central-Western Amazonia, (ii) Northeastern Amazonia, and (iii) Cerrado and Atlantic Forest domains (Fig. 1). However, a second STRUCTURE run within these major clusters revealed a finer pattern of population and lineage subdivision and identified a total of nine groups that were strongly supported according to the ΔK method and log P(X|K). RAxML and ExaBayes species trees were compatible with the STRUCTURE results, and all nine genetic clusters are monophyletic. SVDquartet and BPP (A01 analysis) also strongly support the monophyly of nine genetic clusters (posterior probability > 0.99). In addition, the BPP species delimitation analysis supported a nine-species model with posterior probabilities between 0.72–0.75 and an eight-species model with posterior probabilities around 0.17–0.20 regardless of the prior settings used.

Morphological analysis suggests that at least six distinct groups can be resolved based on normal mixture models (NMM) assuming 1 to 10 distinct morphological groups. The NMM results indicated a best-fit model with six distinct morphological groups (Figure S1). Nonetheless, models set for seven and eight species had similar but slightly lower ΔBIC-values. These groups can be distinguished based on several key criteria that have been recognized by the taxonomic specialists in *Protium* to delimit species (Table S1). The lineages clustered by STRUCTURE can be distinguished by both vegetative traits (e.g., plant habit, number of juga, leaflet size and shape, venation, leaflet thickness) and reproductive traits (inflorescence shape, flower density, petal shape and size, petal coloration, anthers and pistil size (See Table S2 and Figure S2 for morphological details and photos).

Based on the morphological disparity and the genetic clustering evidence, we conclude that *P. heptaphyllum s.l*. represents eight evolutionary lineages that warrant treatment as separate species. Although lineages from the Central Brazil Cerrado (light blue, Fig. 1) and the Atlantic Coast (dark blue) are morphologically distinct by key vegetative and reproductive traits, there is evidence for partial genetic admixture among these clades according on the STRUCTURE analysis. In addition, the BPP species delimitation inferred an eight-species scenario with lower but extant posterior probability mean of 0.18. Therefore, we opted for a conservative delimitation decision in which the Cerrado and Atlantic Forest groups are classified as a single taxon due to evidence of admixture and potential geneflow among lineages. Many of these putatively new lineages are geographically restricted and habitat specialists. When overlaying the geographic ranges of these lineages onto the density of *Protium heptaphyllum s.l*. from the Amazon Tree Diversity Network (ATDN, http://atdn.myspecies.info/; Fig. 1), it is probable that trees listed as *Protium heptaphyllum* in different areas are likely to be assigned to many of the putative new taxa described in Figure S2 and Table S2.

### Demographic history and genetic differentiation

**Figure 2:**
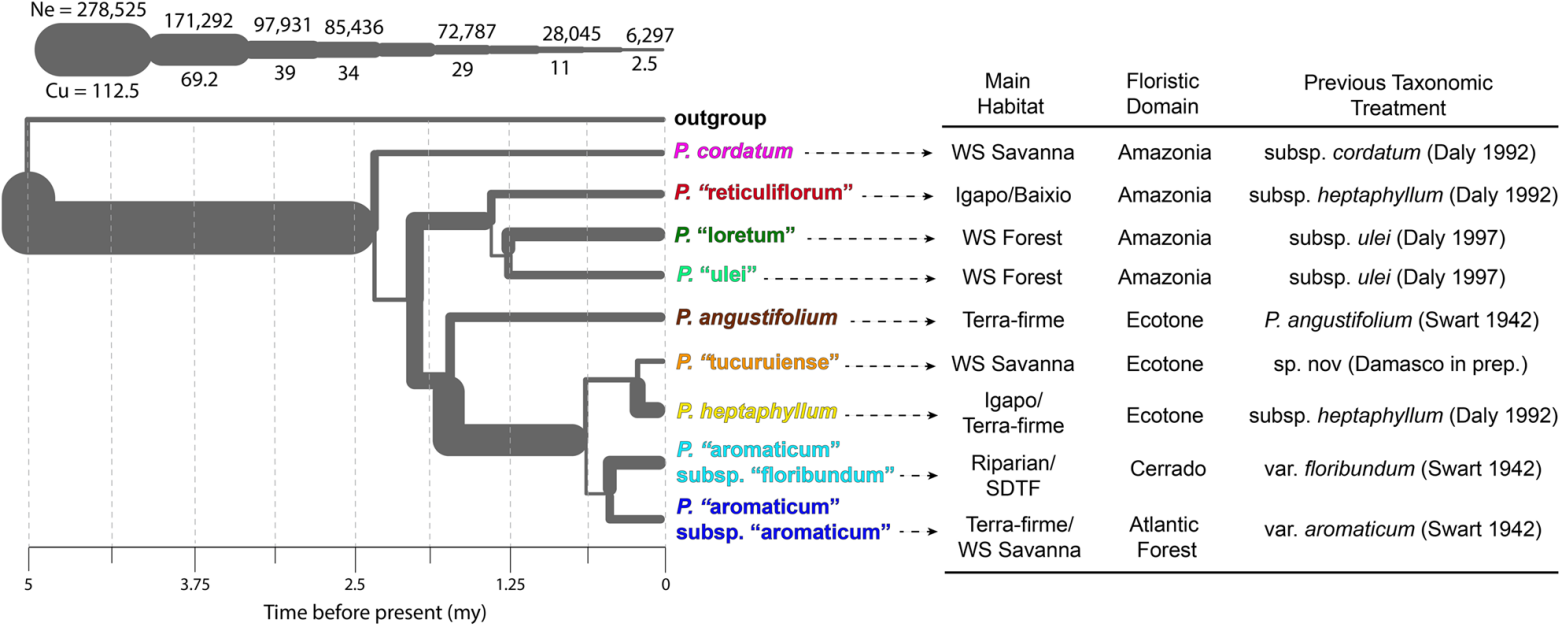
Historical demography and divergence estimate of *Protium heptaphyllum s.l*. populations. Effective population sizes (Ne) based on coalescent units (Cu), and divergence times estimated using the Generalized Phylogenetic Coalescent Sampler (G-PhoCS). The branch ranges (or width) correspond to 95% Bayesian credible intervals aggregated across three runs. Tip labels and colors represent the genetic population assignment based on STRUCTURE analysis. The plant names in quotes are informal, and they will be published in a separate taxonomic review of *P. heptaphyllum s.l*.. On the right, the table displays the habitat type, floristic domain, and the previous taxonomic treatment for each lineage. The floristic domain “Ecotone” corresponds to populations located in transitional areas between the Amazonian floristic domains and neighboring biome areas (e.g. Dry Forests and Grasslands in Colombia). Tree topology generated in FigTree version 1.1 (http://tree.bio.ed.ac.uk/software/figtree/) and branch thickness varies accordingly to Ne and Cu values.

**Figure 3:**
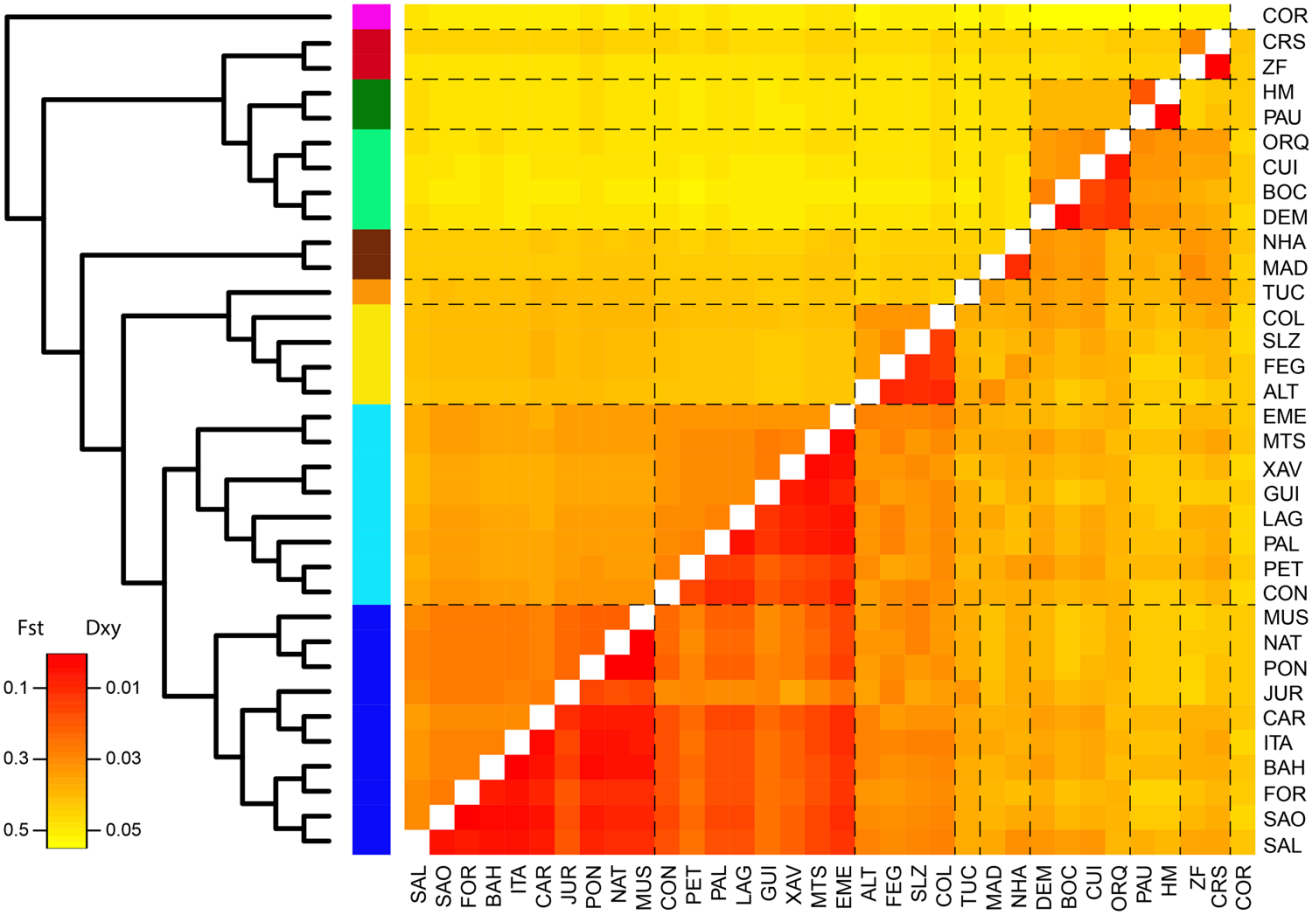
Genetic differentiation among populations of *Protium heptaphyllum s.l*. Pair-wise genetic distances among *P. heptaphyllum s.l*. populations based on two metrics: F_ST_ (lower matrix), a relative measure of genetic differentiation within and between pair-wise populations, and D_xy_ (upper matrix), an absolute measure of genetic divergence between pair-wise populations. Colored bars correspond to populations assignment based on the STRUCTURE analysis. Heatmap generated using heatmap function in RStudio version 1.1.447 (https://rstudio.com/products/rstudio/).

G-PhoCS estimates of divergence time and effective population sizes suggest that *Protium heptaphyllum s.l*. have experienced several distinct trajectories of demographic expansion. *P. heptaphyllum* populations diverged from the outgroup clade ca. 5 Ma after a relative increase in population size (Fig. 2). According to divergence time results, the earliest-diverging *P. heptaphyllum* lineages evolved in the Amazon basin and currently occur in white-sand forests and seasonally flooded habitats. On the other hand, late-diverging lineages diversified very recently, ca. 0.6 Ma, throughout the tropical rain forests and seasonally dry habitats from Central Brazil and the Atlantic Forest. Besides representing multiple distinct species in Amazonia, *P. heptaphyllum s.l*. shows contrasting values of current effective population sizes. In terms of genetic differentiation, F_ST_ and D_xy_ metrics indicate high similarity and potential gene flow among relatively recently diverged lineages from the Cerrado and Atlantic Forest (dark and light blue lineages, Fig. 3). As lineages become less phylogenetically related and spatially distant, F_ST_ and D_xy_ values increase, suggesting high genetic differentiation between Amazonian populations and more recently-diverged lineages from Central Brazil and the Atlantic coast.

### Functional variation and environmental response

**Figure 4:**
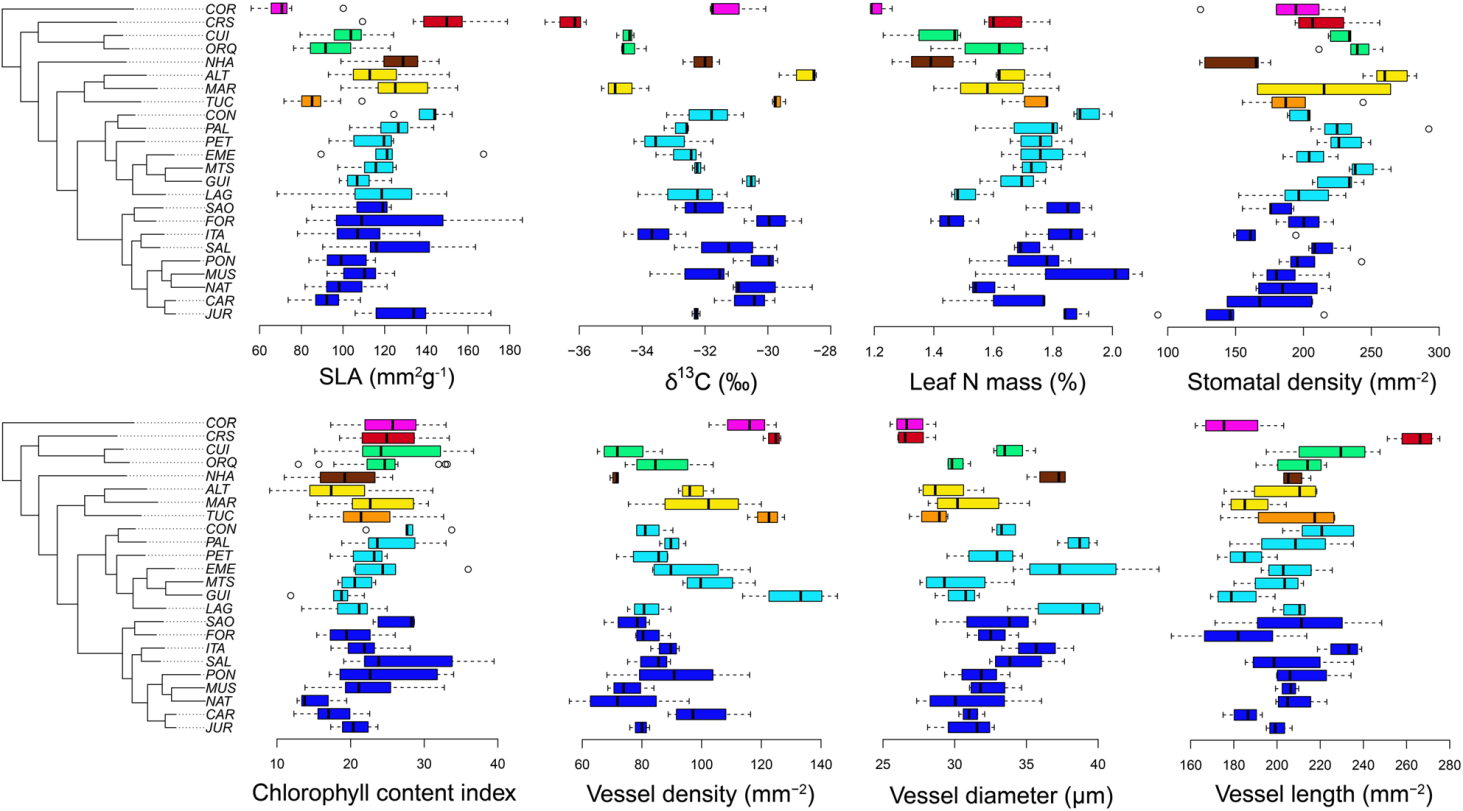
High trait variability among different populations of *Protium heptaphyllum s.l*. **(A)** Leaf and wood trait variation for multiple populations of *P. heptaphyllum s.l.* and color-coded by the genetic clusters identified by STRUCTURE analysis. The results confirm high variability within each population and among putatively discovered lineages. Functional traits were mapped onto a collapsed phylogeny including 24 geographically widespread populations. See Table S3 for complementary details about individual samples and Fig. 1 to access their geographic location. Boxplot graphs generated in RStudio version 1.1.447 (https://rstudio.com/products/rstudio/).

We found that *P. heptaphyllum s.l*. exhibited a complex and variable profile of functional trait characters with clear functional differences in mean trait values and trait breadth among lineages identified by the morphological and molecular analyses (Fig. 4). Lineages from the Amazon region occupying white-sand ecosystems and flooded *igapó* forests had very narrow trait variation. For example, the TUC (orange) and CRS (red) populations exhibit very low within-lineage variation in most functional traits (Fig. 4). In contrast, populations from Central Brazil Cerrado (light blue) and Atlantic Forest (dark blue) often showed greater intra-populational trait variation corresponding to the different habitats the trees inhabit. For instance, *P. heptaphyllum* populations from the Atlantic Forest were commonly sampled across forest-savanna ecotones (e.g. rainforest and *restinga* transect gradients, populations with code MUS and PON, respectively) and leaf and habit traits in these populations exhibited phenotypic plasticity, corresponding to the local soil fertility and water availability (i.e. stunted trees or shrubs less than 2 m tall inhabiting dry and poor soils known as coastal *restingas,* and large 30 m trees occupying nutrient-rich and moister soils in the neighboring forest habitat).

The functional differences we observed among lineages are consistent with differential responses across heterogeneous environments. Overall, functional trait variability among different populations was significantly correlated with local climatic and soil conditions (Table 1). Leaf traits were mostly influenced by the precipitation in the wettest period, and wood traits were well correlated with precipitation deficit in the dry season. In addition, leaf and wood functional traits appear to be directly influenced by local soil conditions, including base saturation, cation exchange capacity and clay content (Table 1).

**Table 1:** Leaf and wood functional traits are influenced by climatic and soil conditions.

	SLA (mm^2^ g^−1^)	δ^13^C (‰)	Leaf N (%)	CCI	Stomatal Dens (mm^−2^)	Vessel Diam (µm)	Vessel Dens (mm^−2^)	Vessel length (µm)
**Bioclimatic variables**								
Mean diurnal range (max. temp.—min. temp)	0.156	0.017	0.212	−0.191	0.150	0.083	0.008	−0.139
Precipitation of driest quarter	−0.206	−0.187	0.000	0.120	0.271	−0.006	−0.174	0.485***
Precipitation coefficient of variation	0.201	−0.029	−0.107	−0.140	−0.063	0.049	0.078	−0.451**
Annual precipitation	−0.300**	−0.369**	−0.319**	−0.028	0.306	0.105	0.146	0.255
Precipitation of driest month	−0.152	−0.211	0.057	0.111	0.261	0.004	−0.209	0.523***
Precipitation of wettest month	−0.286	−0.220	−0.582****	−0.229	0.113	0.011	0.155	−0.135
**Soil variables**								
Calcium (mmol dm^−3^)	−0.129	0.364*	−0.136	−0.232	−0.233	0.371*	−0.129	0.204
Magnesium (mmol dm^−3^)	−0.302*	0.239	0.001	−0.027	0.021	0.304*	0.282	0.273
Carbon (%)	0.252	−0.226	−0.247	−0.469***	−0.492**	0.554****	0.099	0.210
Base saturation (%)	−0.287	0.385**	0.063	−0.127	0.001	0.193	0.084	0.208
Cation exchange capacity (mmol dm^−3^)	0.197	−0.107	−0.319*	−0.295***	−0.490*	0.229	0.158	0.062
Clay content (%)	−0.201	−0.232	0.137	0.095	0.026	0.611****	0.065	0.373**

Linear correlation among phylogenetically independent contrasts of leaf and wood functional traits and their respective bioclimatic and soil variables. Functional traits were measured in 24 geographically widespread populations. Bioclimatic values were extracted from the Worldclim dataset and soil features were obtained from chemical analysis. Significant correlations bolded (* *P* < 0.05; ** *P* < 0.01; *** *P* < 0.001; **** *P* < 0.0001).

*SLA* specific leaf area, *δ*^*13*^*C* carbon stable isotopes, *N* nitrogen, *CCI* chlorophyll content index, *Dens.* density, *Diam.* diameter.

## Discussion

Based on extensive geographic sampling of morphological, ddRADseq and functional trait data, we have identified eight distinct evolutionary lineages within the putative hyperdominant taxon *P. heptaphyllum*. We are using a “traditional” species concept—a typological or morphological species concept where we expect different species to have discrete morphological differences but also represent monophyletic groups with limited genetic admixture. Morphological traits overlap at varying levels, but genetic data suggest that this widespread group represents distinct, independently adapted lineages that diverged over the past million years. We refute the hypothesis that *P. heptaphyllum s.l*. is a single species and instead find evidence for recognizing eight independently evolving species (or lineages, *sensu*^20^). Updates regarding the taxonomic treatment within *P. heptaphyllum s.l.* are in progress (e.g. *Protium cordatum* Huber *sensu*^21^) and detailed descriptions of new species are in preparation as part of a taxonomic revision (Table S2, Figure S2).

Even though a large number of new species have been recently described in the Neotropics^9,10,22^, very few studies have attempted to resolve morphologically challenging species complexes (e.g.,^13,15^). In the sections below, we discuss the implications of our results in the context of: (i) revisiting the concept of hyperdominance for Amazonian trees, (ii) improving richness and diversity estimates, (iii) understanding diversification within dominant tropical lineages, and (iv) refining predictions for ecosystem function.

### Implications for the hyperdominance phenomenon

The fact that communities often harbor a small group of demographically abundant species in addition to a much larger number of rare species is not a recent discovery (e.g.,^23^ as cited in^3^). This pattern, also called a species oligarchy and hyperdominance, was first reported for western Amazonian forests in the early 2000s^24,25^ and again on the pan-Amazonian scale based on data from the ATDN, one of the largest tree community datasets ever compiled in the tropics^1^. Hyperdominant species have captured the imagination of many tropical ecologists. First, they have been thought to be more likely to be correctly identified than rare species^22^, allowing for people to use them as proxies for ecosystem-wide function. For example, Fauset et al.^4^ emphasized that hyperdominant species were responsible for half of carbon storage and productivity in the Amazon. Second, ecologists have proposed that hyperdominants have important shared demographic properties—they often have large geographic ranges but are only dominant in one or two regions and are often habitat specialists^1^. In contrast, our results suggest that the hyperdominant taxon *P. heptaphyllum s.l.* actually consists of several lineages warranting recognition as new species that have very different functional traits, that occupy distinct geographic ranges, and that can be rare or threatened. We postulate that similar conclusions could be reached with many of the other hyperdominant species, which are also thought to be members of species complexes (e.g., *Iriartea deltoidea* Ruiz and Pav.^26^; *Eschweilera coriacea* (DC.) S.A. Mori^27^). Further study is warranted prioritizing the study of these putative species complexes to refine our understanding of dominance across tropical regions^28^. If fewer species are found to be true hyperdominants, this will render conservation efforts and ecosystem modeling exercises more complicated than has been discussed to date.

### Taxonomic relevance

*Protium* is one of the best studied plant groups in the Neotropics (e.g.,^17–19^). Currently, the genus consists of approximately 200 species, and their systematics has been studied by a collaborative team of taxonomists and evolutionary biologists. *Protium* has a wide geographic range of specimen sampling, and genomic data are available for a number of species^18,29,30^. Also, current species descriptions in *Protium* are consistently founded on both morphological and molecular phylogenetic evidence^19^. Furthermore, the broad intraspecific sampling of key lineages/taxa within *Protium heptaphyllum s.l.* gives us high confidence in our results, in contrast to other plant groups that have not yet been subjected to similar intensive systematic study but often include diverse and abundant trees in the Amazon Basin. Here, we showed that a multidisciplinary effort and relatively short time investment on a hyperdominant species complex (3–5 years) has yielded the discovery of eight new lineages warranting species status.

Multiple other tree families are relatively well studied in the Neotropics (e.g., Annonaceae, Sapotaceae, Lecythidaceae, Rubiaceae, Chrysobalanaceae, Fabaceae, Melastomataceae), but are also frequently cited as containing a few species complexes with incomplete genetic divergence and requiring detailed revision (e.g., *Eschweilera*^27^, *Pouteria*^31^). We acknowledge that the field of taxonomy is dynamic (ter Steege et al. 2019) in the sense that classification and species names are likely to change as new studies are performed. Besides that, the methods applied to describe, re-establish, or invalidate taxonomic entities are frequently inconsistent among taxonomists and experts, and can vary according to several species concepts and definitions^32,33^. Inconsistency in taxonomic methods has been the focus of recent debates about tree species richness in Amazonia^9,10^. While the authors of these studies disagreed about the number of Amazonian tree species, both sides agree that there is still much work to be done in order to improve taxonomy and to increase the pace of species discovery^22^. We propose that the establishment of a consensus prioritized list of other species complexes from putative hyperdominant taxa such as *P. heptaphyllum, s.l.* would represent a significant step to contribute to efficient progress of new species discovery. With the advent of cheaper and more rapid techniques for both molecular systematics and functional ecology, we expect that interdisciplinary approaches combined with an extensive populational sampling like we have done will be highly beneficial for the study of hyperdominant species complexes and to advance the estimates of Amazonian tree species diversity.

### Refining the understanding of trait variation and functional response

The seven distinct lineages for the Central/West Amazon Basin and Northeast Amazonian regions showed substantial variation in leaf and wood traits (Fig. 4). Overall, more than half of the breadth of variation in these functional traits that has been observed across 2600 Amazonian species (e.g.,^34–36^) can be found within this single species complex.

Moreover, the breadth of functional trait variation also varied markedly within lineages. The low within-lineage trait variation in the Amazonian TUC (orange) population (Fig. 4) is consistent with adaptation to dry savanna environments; that is, this lineage exhibits consistent high-water use efficiency (less negative 13C) and small, dense xylem vessels. In contrast, the CRS (red) population, which also exhibits low within-lineage functional trait variation, displays values of traits consistent with functional responses to flooding in Amazonian *igapó* habitats where it is found, and thus has low water use efficiency and large xylem vessels. Together, these lineages contribute to the broad variation observed in the species complex, while at the same time other lineages from Central Brazil and the Atlantic Coast (light and dark blue) show high within-lineage functional trait variation and broad distribution across habitats. Taken together across the phylogeny of the entire species complex, these functional traits can be considered to be highly labile, with some lineages adapting to contrasting extreme values whereas other sister lineages retain broad functional trait variation (Fig. 4).

Importantly, this variation is consistent with the evolutionary histories of each distinct lineage within the species complex. For example, the *P. heptaphyllum*, *P.* “tucuruiense” and *P.* “aromaticum” (yellow, orange and blue) clades experienced a relatively recent population bottleneck and then expanded, likely with traits that permitted them to radiate into the drier savanna environments where they were then able to expand (Fig. 2). Some of these lineages have become adapted to distinct habitats and are responding very differently to current environmental conditions. Moreover, we suggest that these lineages will respond very differently to future changes in climatic conditions across the region, with some of them poorly equipped for the predicted increasing frequency and intensity of droughts^37^.

Our results have important implications for ecosystem function. Since the advent of functional trait network studies (e.g., TRY Plant Trait Database^38^), understanding how plant species behave in terms of their physiological performance over large scales has led to important predictions of the consequences of future scenarios of climate and land-use change^39–41^. The importance of the Amazon region for the global climate and carbon cycle^42,43^ highlights the need to devote substantial effort to investigating the taxonomy of hyperdominant plant taxa. For instance, if some or most of the hyperdominant taxa actually represent multiple hidden evolutionary entities, as in *P. heptaphyllum s.l*., a larger fraction of Amazonian tree species would contribute proportionally more to carbon storage and cycling than described by Fauset et al.^4^, rendering modeling exercises and management much more nuanced. In addition, a comprehensive taxonomic review of dominant tree lineages with concomitant screening of functional traits as we conducted for this species complex, would greatly improve the understanding of climate-induced functional shifts, such as described by Esquivel‐Muelbert et al.^41^, and have potentially important consequences for the conservation of putative rare taxa that are nested within hyperdominant species complexes.

### Understanding diversification in dominant lineages

The scenario of some hyperdominant or oligarchic taxa representing multiple diverged lineages is intriguing and relevant for understanding the processes of diversification in the Amazonian flora. Based on the demographic history of *P. heptaphyllum s.l.*, older lineages tend to be habitat specialists and less morphologically and functionally variable. In contrast, recently evolved lineages have colonized new areas and frequently experience gene flow across very large geographic distances, and they are morphologically and functionally more variable. We hypothesize that large population sizes may be associated with higher diversification rates due to the process of population expansion followed by radiation into different habitats. Therefore, dominant lineages would have better chances to speciate via habitat specialization and generate large clades than non-dominant lineages. Habitat specialization is considered to have evolved in many tropical plant groups^44,45^ and many different tree genera have become specialized in contrasting environments^46^.

According to our results, *P. heptaphyllum s.l*. diverged from its common ancestors around five million years ago and diversified first in the Amazon region followed by an increase in population size. Many lineages subsequently became specialized to white-sand habitats, seasonally flooded forests in the Rio Negro Basin (*Igapó* forests), and floodplain forests (*várzea* forests). However, more recently, lineages dispersed into neighboring floristic domains (e.g. Cerrado and Atlantic Forest) and ecotone areas, colonizing regions where the annual precipitation is currently lower and seasonal. These populations found in Central and Coastal Brazil are genetically very similar to each other despite the large geographical distances among them. Moreover, these populations are functionally more variable than the early-diverging lineages in the Amazon. In contrast, older lineages from the Amazon basin were found to be less morphologically plastic and more isolated in terms of gene flow. White-sand ecosystems in Amazonia are characterized by a patchy and geographically disjunct distribution^47^ that could have inhibited dispersal of habitat-specialist populations, resulting in repeated speciation in white-sand forests in different regions.

### Conservation implications

We show that at least four newly discovered lineages, including two “resurrected” species, are geographically restricted, demographically rare and endemic to white-sand vegetation in the Amazon (e.g. *P. cordatum *sensu Damasco et al. 2019a*, P.* “tucuruiense*,” P. angustifolium* Swart*,* and *P.* “reticuliflorum”). Future studies aiming to review potential species complexes among hyperdominant species could indicate other threatened lineages warranting taxonomic recognition as well as new geographic areas for conservation priority. While many studies have relied on datasets compiled by plot-inventory networks, more effort should be focused on increasing the pace of taxonomic research in the Neotropics^22,28^. Deforestation rates in Amazonia are likely to increase in the next few years; and yet one third of the total number of tree species are likely still undescribed or undiscovered^1^.

We believe that reports that roughly half of all biomass and carbon storage in one of the most diverse areas on Earth is stored by a very small group of “hyperdominant” species may be misinforming policy makers and stakeholders. Based on our integrative review of *P. heptaphyllum s.l*., we showed that genetic diversity and functional responses to environmental gradients are much greater than expected by the hyperdominance principle. Our results revealed that a single “hyperdominant” taxon contains several lineages warranting recognition as distinct species that include great functional diversity, including at least three that are relatively rare and potentially threatened. Metapopulations of a hyperdominant taxon may be interpreted as much more resilient to future global changes than relatively rare lineages within a species complex. Thus, our findings suggest not only critical issues with species level conservation for lineages that should now be considered threatened taxa and not hyper- nor dominant, but also that some of these putatively new lineages might be at risk of extinction due to future climate change and increasing deforestation. Although the distinction between species and population delimitation may not be of consequence for calculating current carbon storage, we argue that multiple distinct lineages with limited gene flow such as we describe here will likely respond very differently to current and future global changes than a larger interbreeding metapopulation of a single lineage. We therefore caution against simplistic assumptions about biogeochemical processes and ecosystem services reliant on the attractive simplicity of a putative hyperdominance phenomenon.

## Methods

### Field data collection

We conducted multiple field sampling across the geographic range of *Protium heptaphyllum s.l.* in South America (Fig. 1; Table S3). In total, 39 populations of *P. heptaphyllum* were sampled, in addition to ten closely related outgroup species selected based on a molecular phylogeny of the Protieae tribe^18^, i.e., *P. unifoliolatum*, *P. trifoliolatum*, *P. krukoffii*, *P. pillosum*, *P. widgrenii*, *P. icicariba*, *P. kleinii*, *P. brasiliense*, *P. ovatum,* and *P. dawsonii* (Table S3). Outgroup taxa were sampled in the field when possible; otherwise, herbarium specimens were used. For each sample, we recorded latitude, longitude, and elevation with a GPS device. Leaf material for DNA extraction was dried right after collection using silica gel and subsequently stored in a − 20 °C freezer. In addition, we performed leaf morphological measurements, extracted DNA, and collected functional trait data on the same plant specimens. Soil texture and fertility was analyzed from each population site. Morphological, ddRAD sequences, and functional trait data are provided in the supporting information. Fertile voucher specimens are in preparation to be deposited at the New York Botanical Garden (NY), the University Herbarium (UC) and *Instituto Nacional de Pesquisas da Amazonia* (INPA). Experimental research on plants, including the collection of plant material, comply with the relevant institutional, national, and international guidelines and legislation. Collections permits were provided by SISBIO (Biodiversity Authorization and Information System) in Brazil (document numbers 37726-3, 44650-1 and 48657-1). Formal identification of the plant material used in this study was performed by Douglas C. Daly, Gabriel Damasco, Paul V. A. Fine and Ricardo O. Perdiz.

### Morphological measurements

We generated a character matrix with 59 continuous and 98 discrete traits (supplemental Table S1) to investigate the variability of morphological characters in a multidimensional space. Non-informative characters and missing data were excluded. We used the R package clustvarsel v.2.3.3^48^ to reduce the dimensionality of the data by selecting the set of principal components most useful for discrimination without a priori grouping information. Vegetative and reproductive traits were measured on 104 specimens of *P. heptaphyllum s.l*.. To test the hypothesis that *P. heptaphyllum s.l*. represents multiple lineages worthy of taxonomic recognition, we fit the number of morphological clusters using the normal mixture models (NMMs) implemented in the R package mclust v.5.0^49^. The Bayesian information criterion (BIC^50^) was used to evaluate the best-fit number of morphological groups according to each NMM^51^.

### DNA extraction and ddRAD library preparation

We extracted high-quality genomic DNA from 385 samples of *P. heptaphyllum s.l*. widely distributed throughout the Amazon, Atlantic Forest, and Cerrado, and six outgroup species (see Table S3 for details about specimens). DNA was extracted from ca. 100 mg of leaf tissue preserved in silica or from herbarium specimens when silica-dried leaves were not available. Extractions followed an updated version of the DNEasy Plant mini kit protocol (Qiagen, Crawley, U.K.). Double-digest RAD-seq libraries (paired-end length of 180 bp) were prepared for high-throughput sequencing following Peterson et al.^52^ and DNA was digested with *Sph*I-HF and *Eco*RI-HF enzymes. DNA libraries were sequenced on five lanes of an Illumina HiSeq 4000 at the University of Berkeley QB3 facility.

### ddRAD data analysis

We assembled the ddRAD-seq reads using the software ipyrad version 0.6.17^53^, in order to generate two de novo assemblies with different levels of sample sizes and missing data. First, we used a clustering threshold of sequence similarity set to 0.85 and retained a data set including all loci shared by at least four samples. All other ipyrad parameters were set to default. In total, 23,922 loci and 39,770 SNPs were recovered from 385 samples (dataset 1). Although the average sample coverage was high (385 loci per sample), 56% of samples had high levels of missing data. We generated another assembly using a higher cluster threshold of sequence similarity (0.90) and including all loci shared by at least 10 samples. The low-missing-data assembly resulted in 6234 total filtered loci and 25,027 SNPs from 285 samples, and 21% missing data (dataset 2).

### Genetic differentiation

To estimate the number of genetic clusters within *P. heptaphyllum s.l*. without a priori assumptions of individual assignments, we used the Bayesian clustering algorithm STRUCTURE^54^ on dataset 1. We ran 10 replicates at each value of *K* for 500,000 generations with a burn-in of 50,000. *K* = 3 was preferred with the ΔK method and had the highest log P(X|K). Those genetic clusters corresponded with the populations located in three major biogeographic regions: (i) the Amazon biome, (ii) ecotone zones between the Amazon and neighboring biomes, and (iii) a large geographic region encompassing the Cerrado and Atlantic Forest biomes (Fig. 1). In order to identify finer patterns of genetic structure, we ran a separate analysis within the three identified groups using similar parameter settings. We used Structure Harvester version 0.6.94 to compare alternative values of K based on the log probability (log P(X|K)) and the ΔK statistic^55^. Pairwise F_ST_ and D_XY_ were calculated among populations using dataset 1. F_ST_ was implemented based on the Weir and Cockerham equations^56^ found in the WCfst function from the hierfstat R package^57^. D_XY_ was calculated as described by Nei^58^, with a custom R script slightly modified from the genet.dist function in hierfstat. Among different lineages, F_ST_ and D_XY_ values ranged from 0.1 to 0.43 and 0.028 to 0.055, respectively. In contrast, within same lineages, F_ST_ and D_XY_ values ranged from 0.08 to 0.19 and 0.021 to 0.032, respectively. D_XY_ measures absolute genetic distance, while F_ST_ measures genetic differentiation among populations relative to within populations^58^. F_ST_ values under 0.19 (or 0.20) are usually interpreted as lacking significant genetic structuring or population subdivision. At the same time, F_ST_ values above 0.3 are seen as moderate population structuring. F_ST_ values above 0.5 are normally considered to be associated with strong population subdivision.

### Divergence time and population size estimates

The demographic parameters were implemented in the Generalized Phylogenetic Coalescent Sampler (G-PhoCS version 1.2.3^59^), a Bayesian method for inferring divergence times and effective population sizes from genome sequences. First, we generated a de novo assembly with 1971 loci and 43 samples selected from populations within the 10 different clusters defined by STRUCTURE and a collapsed clade of closely related outgroups. In the MCMC runs, we used the gamma distribution with α = 1.0 and β = 10,000 for the mutation-scaled population sizes and divergence times, and a gamma distribution with α = 0.002 and β = 0.00001 for the mutation-scaled migration rates. Each Markov Chain was run for 100,000 burn-in iterations, after which parameter values were sampled for 500,000 iterations every 50 iterations. Convergence was inspected manually for each run. Parameter calibration in the probabilistic model of G-PhoCS is scaled by mutation rate μ. We estimated an average mutation rate of μ = 7.2 × 10^–5^ mutations per site per generation given by τ = *T*μ/*g*, where τ is the parameter tau, *g* is the average generation time (in years), and *T* is the absolute divergence time (in years). We used a divergence time of 5 Ma (95% CI: 3.8–11.1 Ma) for the clade including *P. heptaphyllum* and close relative outgroups based on Fine et al.^18^ and the generation time of ten years was based on an estimation of the average reproductive age of many individuals observed in the field. Effective population sizes are given by θ = 4*N*_*e*_μ, where θ is parameter theta and *N*_*e*_ is the absolute effective population size, and the migration rates are given by *M* = *m*/μ, where *m* is the probability of migration across two given populations.

### Phylogenetic reconstruction

To reconstruct phylogenetic relationships among individuals we took multiple approaches. Trees were inferred from concatenated data sets using RAxML version 8.2.10^60^ and ExaBayes version 1.5^61^ on dataset 2. For RAxML, maximum likelihood phylogenies were inferred with 1000 bootstraps using the GTRGAMMA substitution model. For ExaBayes analysis, we ran two Metropolis-coupling replicates with four coupled-chains (each with three heated chains) for 1 × 106 MCMC generations, sampled every 500 generations. Estimated sample size (ESS) for all parameters and branch lengths were summarized with the postProcParam tool and confirmed as sufficiently sampled after analyzing in Tracer v.1.6^61^. Finally, we generated a majority-rule consensus tree with the Exabayes ‘consense’ tool after a 25% burn-in. Each tree was rooted by the clade composed of *P. unifoliolatum*, *P. trifoliolatum*, *P. krukoffii*, *P. brasiliense*, *P. icicariba*, and *P. widgrenii*.

### Species tree inference and species delimitation

We inferred species trees on dataset 2 using the coalescent-based methods SVDquartet^62^ conducted in PAUP 4a157 (http://phylosolutions.com/paup-test/), and BPP version 3.4^63^. The data were scored with different populations set as partitions using the A01 analysis^64^. For SVDquartet, we used default settings except for the species tree option and conducted 1000 bootstrap replicates. The posterior distribution was independent of different starting species trees and the topology was consistent when comparing results of multiple runs. We used the A10 analysis in BPP version 3.4 to test species delimitation within the *P. heptaphyllum s.l*. group using a fixed species tree previously inferred by SVDquartet and BPP. We assigned equal prior probabilities to all species delimitation models (1 to 10 species) and all runs were sampled every 50 generations for 10,000 samples with a burn-in of 2,000. We evaluated convergence by comparing results of replicate runs. In BPP, we assigned equal probabilities for the rooted trees and set the inverse-gamma priors θ ∼ IG (2, 50) for all θs and τ ∼ IG (4, 50) for the age of the root (τ0) according to the demographic inference previously performed by G-PhoCS analysis.

### Functional traits

In order to understand the physiological responses of distinct *P. heptaphyllum s.l*. populations across different climatic and environmental conditions, we measured multiple functional characters including leaf, wood, and chemical traits. The leaf traits measured were specific leaf area (SLA), leaf nitrogen content, leaf carbon content, leaf stable carbon isotope (13C) composition, leaf chlorophyll content, leaf stomatal density. The anatomical wood traits were vessel diameter, vessel density, and vessel length. The phylogenetic independent contrast (PIC) was calculated for each functional trait using the R package ape version 5.3. Linear correlation between phylogenetically independent contrasts of functional traits were performed to test the relationship with local climatic and soil features. Details about each functional trait is available in Table S4.

## Supplementary Information

Supplementary Figure S1.

Supplementary Figure S2.

Supplementary Table S1.

Supplementary Table S2.

Supplementary Table S3.

Supplementary Table S4.
