# Supplementary material for: Revisiting the hyperdominance of Neotropical tree species under a taxonomic, functional and evolutionary perspective
Source: Sci Rep. 2021 May 5;11:9585. doi: 10.1038/s41598-021-88417-y (PMC8099866; doi:10.1038/s41598-021-88417-y)
Supplement: Supplementary file 1 — Supplementary Figure S1. [file 41598_2021_88417_MOESM1_ESM.docx]

**Revisiting the hyperdominance of Neotropical tree species under a taxonomic, functional and evolutionary perspective**

**Gabriel Damasco^a,b,d^
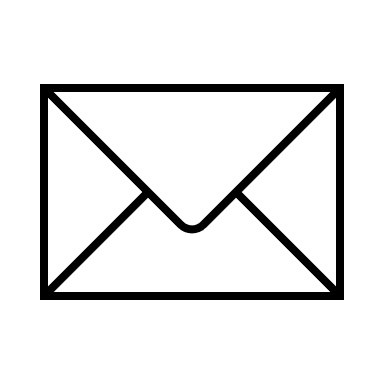
, Christopher Baraloto^c^, Alberto Vicentini^d^, Douglas C. Daly^e^, Bruce G. Baldwin^a^, Paul V. A. Fine^a^**

**^a^** Department of Integrative Biology, University of California, Berkeley, CA 94720-3140, email: [gdamasco@berkeley.edu](mailto:gdamasco@berkeley.edu), [gabrielfloresta@gmail.com](mailto:gabrielfloresta@gmail.com); **^b^** Department of Biology and Environmental Science, University of Gothenburg, Sweden; **^c^** International Center of Tropical Biology, Florida International University, Miami, FL 33133; **^d^** Instituto Nacional de Pesquisas da Amazônia, Programa de Pós–graduação em Ciências Biológicas (Botânica), Manaus, AM 70390‐095; and **^e^** Institute of Systematic Botany, The New York Botanical Garden, Bronx, NY 10458.


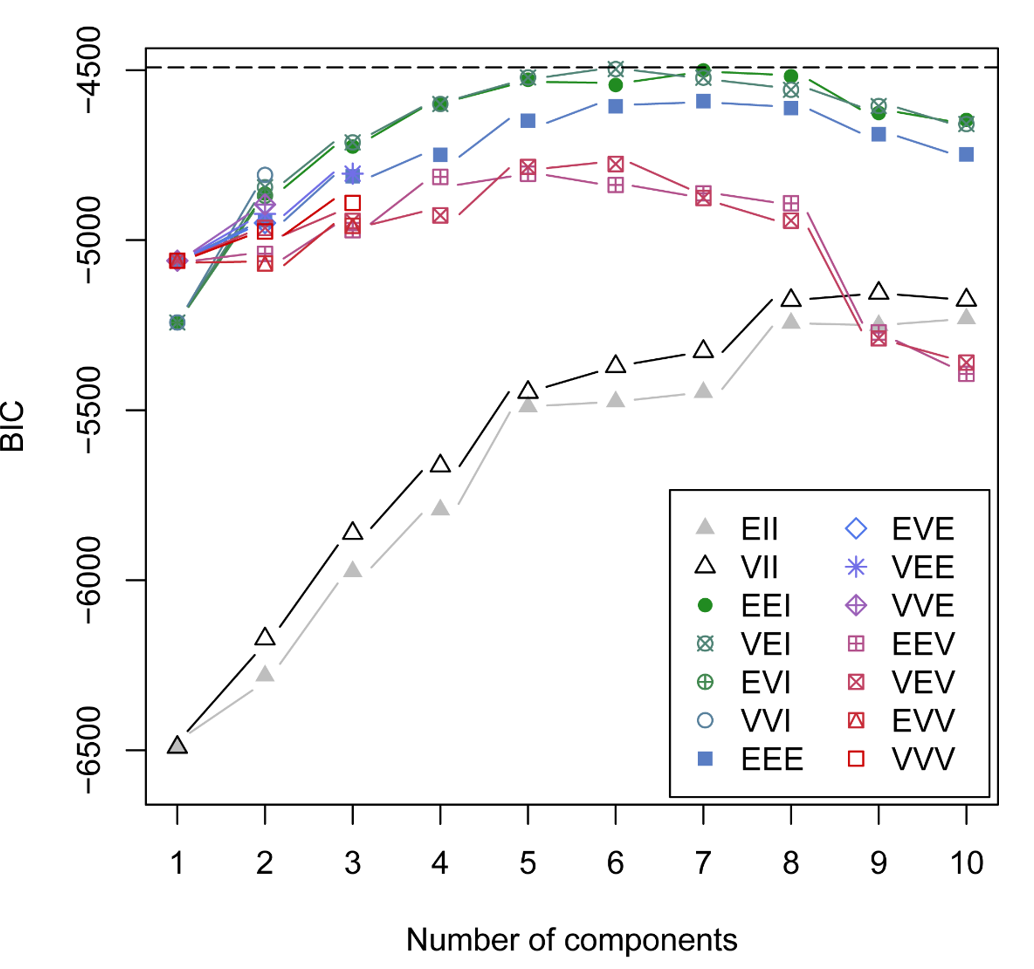


**Fig. S1.** Morphological data strongly supported hypotheses that there are multiple distinct groups within *Protium heptaphyllum s.l.* The graph shows the support for normal mixture models (NMM) assuming 1-10 distinct morphological groups and 12 model parameterizations. The two models with highest support based on BIC values assumed six and seven distinct morphological groups (models EEI and VEI).
