# Supplementary material for: Revisiting the hyperdominance of Neotropical tree species under a taxonomic, functional and evolutionary perspective
Source: Sci Rep. 2021 May 5;11:9585. doi: 10.1038/s41598-021-88417-y (PMC8099866; doi:10.1038/s41598-021-88417-y)
Supplement: Supplementary file 3 — Supplementary Table S1. [file 41598_2021_88417_MOESM3_ESM.docx]

**Revisiting the hyperdominance of Neotropical tree species under a taxonomic, functional and evolutionary perspective**

**Gabriel Damasco^a,b^
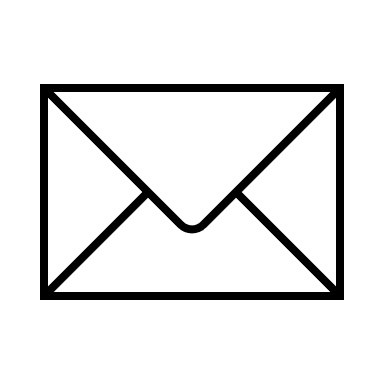
, Christopher Baraloto^c^, Alberto Vicentini^d^, Douglas C. Daly^e^, Bruce G. Baldwin^a^, Paul V. A. Fine^a^**

**^a^** Department of Integrative Biology, University of California, Berkeley, CA 94720-3140, email: [gdamasco@berkeley.edu](mailto:gdamasco@berkeley.edu), [gabrielfloresta@gmail.com](mailto:gabrielfloresta@gmail.com); **^b^** Department of Biology and Environmental Science, University of Gothenburg, St. Paul, MN 55108; **^c^** International Center of Tropical Biology, Florida International University, Miami, FL 33133; **^d^** Instituto Nacional de Pesquisas da Amazônia, Programa de Pós–graduação em Ciências Biológicas (Botânica), Manaus, AM 70390‐095; and **^e^** Institute of Systematic Botany, The New York Botanical Garden, Bronx, NY 10458.

**Table S1.** Selected morphological characters consistently used in taxonomic studies in Burseraceae.

| Plant Part | Trait | Data type |
| --- | --- | --- |
| Leaf | Leaf length total (min/max) | continuous |
|  | no. juga | continuous |
| Petiole & rachis | Petiole L x diam. | continuous |
|  | indumentum | discrete |
| Petiolules & pulvinuli | basals L | continuous |
|  | laterals L | continuous |
|  | thickness | continuous |
|  | terminal L | continuous |
|  | canaliculate | discrete |
|  | pulvinuli lateral | continuous |
|  | pulvinulus terminal | continuous |
| Leaflets -- size and shape | basals: L x W | continuous |
|  | laterals: L x W | continuous |
|  | terminal: L x W | continuous |
|  | basal pair shape | discrete |
|  | laterals shape | discrete |
|  | terminal: shape | discrete |
| Leaflets -- apex and base | all: apex shape | discrete |
|  | lateral base | discrete |
|  | lats: base shape | discrete |
| Leaflets | specific leaf area | continuous |
|  | margin | discrete |
|  | dry color | discrete |
|  | thickness | discrete |
| Leaflet venation | 2o vein framework | discrete |
|  | 2o veins - pairs | discrete |
|  | 2o veins - course & insertion | discrete |
|  | 2o veins - angle | discrete |
|  | 2o veins - spacing | discrete |
|  | 2o discolor? | discrete |
|  | inter-2o and epimedial 3o | discrete |
|  | 3o vein fabric | discrete |
|  | 4o venation | discrete |
| Leaflet surface | vein prominence abax. | discrete |
|  | pub.: abax. | discrete |
|  | vein prominence adax. | discrete |
|  | pubescence: adax. | discrete |
|  | leaflet surface -- other | discrete |
| Inflorescences -- all | position | discrete |
|  | type | discrete |
|  | m = f? | discrete |
| Staminate inflorescences | tot. L x diam. | continuous |
|  | indumentum | discrete |
|  | bracts on 1o axes - size | continuous |
|  | bracts on 2o axes - size | continuous |
|  | bracts - shape | discrete |
|  | semi-clasping? | discrete |
|  | bract apex | discrete |
|  | thickness | discrete |
|  | pubescence | discrete |
|  | bracteoles - size | discrete |
|  | bracteoles - shape | discrete |
|  | thickness | discrete |
|  | indumentum | discrete |
|  | flowers (sub-) sessile? | discrete |
|  | pedicel: L x diam. | continuous |
|  | form | discrete |
| Staminate flowers -- no. of parts, length | no. of flower parts | continuous |
|  | total L (m) | continuous |
| Staminate - calyx | K total L x diam. | continuous |
|  | K > disk? | discrete |
|  | K form | discrete |
|  | lobe L x W | continuous |
|  | lobe L relative to K total? | continuous |
|  | lobe shape | discrete |
|  | K indumentum abax | discrete |
| Staminate - corolla | petal L x W | continuous |
|  | petal color | discrete |
|  | C shape | discrete |
|  | C - thickness | discrete |
|  | C - apiculum length & shape | discrete |
|  | petal orientation at anthesis | discrete |
|  | C indumentum abaxial | discrete |
|  | C indumentum adaxial | discrete |
| Staminate - androecium | number of stamens | continuous |
|  | length antesepalous | continuous |
|  | stamens exserted? | discrete |
|  | length antesep anthers | continuous |
|  | length antepetalous | continuous |
|  | length antepet anthers | continuous |
|  | shape anthers | discrete |
|  | filament shape, texture, surface | discrete |
|  | connective pubescence? glandular? | discrete |
| Staminate -- disk & pistillode | disk type | discrete |
|  | disk dimensions | continuous |
|  | disk indumentum | discrete |
|  | pistillode dimensions overall | continuous |
|  | pistillode - ovariodisk shape | discrete |
|  | pistillode - style | continuous |
|  | pistillode - indumentum | discrete |
| Pistillate inflorescences | tot. L x diam. | continuous |
|  | L 2 axes | continuous |
|  | indumentum | discrete |
|  | bracts on 1o axes - size | continuous |
|  | bracts on 2o axes - size | continuous |
|  | bracts - shape | discrete |
|  | semi-clasping? | discrete |
|  | bract apex | continuous |
|  | thickness | discrete |
|  | pubescence | discrete |
|  | bracteoles - size | continuous |
|  | bracteoles - shape | discrete |
|  | thickness | discrete |
|  | indumentum | discrete |
|  | flowers (sub-) sessile? | discrete |
|  | pedicel: L x diam. | continuous |
|  | form | discrete |
| Pistillate flowers | no. of parts | continuous |
| Pistillate flowers | total L (f) | continuous |
| Pistillate - calyx | K total L x diam. | continuous |
|  | K > disk? | discrete |
|  | K form | discrete |
|  | lobe L x W | continuous |
|  | lobe shape | discrete |
|  | K indumentum abax | discrete |
| Pistillate -- corolla | petal L x W | continuous |
|  | petal color | discrete |
|  | C shape | discrete |
|  | C - consistency | discrete |
|  | C - apiculum shape | discrete |
|  | petal orientation | discrete |
|  | C indumentum abaxial | discrete |
|  | C indumentum adaxial | discrete |
|  | margin | discrete |
| Pistillate - androecium | number of staminodes | continuous |
|  | length antesepalous | continuous |
|  | length antesep anthers | continuous |
|  | length antepetalous | continuous |
|  | length antepet anthers | continuous |
|  | shape anthers | discrete |
|  | filament shape, texture, surface | discrete |
|  | connective pubescence? glandular? | discrete |
|  | thecae separated by connective? | discrete |
| Pistillate - gynoecium | disk dimensions | continuous |
|  | disk indumentum | continuous |
|  | pistil dimensions | continuous |
|  | ovary shape and base | discrete |
|  | pistil - style | continuous |
|  | pistil - stigmatal region height | continuous |
|  | pistil - indumentum | discrete |
|  | stigmas - shape | discrete |
|  | stigmas - orientation | discrete |
|  | stigmas - ornamentation | discrete |
|  | infructescence diameter | continuous |
| Fruit -- external | pedicel L x diam. | continuous |
|  | pedicel shape | discrete |
|  | frt ext. color (mature) | discrete |
|  | L x diam dry | continuous |
|  | shape | discrete |
|  | apex | discrete |
|  | base - (sub) stipitate? | discrete |
|  | base - shape | discrete |
|  | surface | discrete |
|  | fruit indumentum | discrete |
