# Supplementary material for: Revisiting the hyperdominance of Neotropical tree species under a taxonomic, functional and evolutionary perspective
Source: Sci Rep. 2021 May 5;11:9585. doi: 10.1038/s41598-021-88417-y (PMC8099866; doi:10.1038/s41598-021-88417-y)
Supplement: Supplementary file 4 — Supplementary Table S2. [file 41598_2021_88417_MOESM4_ESM.docx]

**Revisiting the hyperdominance of Neotropical tree species under a taxonomic, functional and evolutionary perspective**

**Gabriel Damasco^a,b,d^
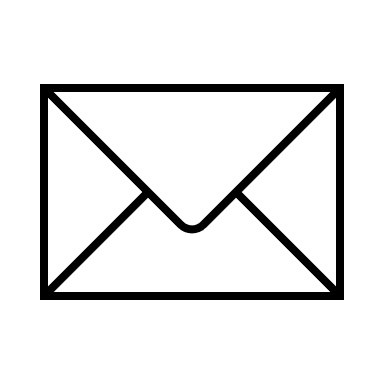
, Christopher Baraloto^c^, Alberto Vicentini^d^, Douglas C. Daly^e^, Bruce G. Baldwin^a^, Paul V. A. Fine^a^**

**^a^** Department of Integrative Biology, University of California, Berkeley, CA 94720-3140, email: [gdamasco@berkeley.edu](mailto:gdamasco@berkeley.edu), [gabrielfloresta@gmail.com](mailto:gabrielfloresta@gmail.com); **^b^** Department of Biology and Environmental Science, University of Gothenburg, Sweden; **^c^** International Center of Tropical Biology, Florida International University, Miami, FL 33133; **^d^** Instituto Nacional de Pesquisas da Amazônia, Programa de Pós–graduação em Ciências Biológicas (Botânica), Manaus, AM 70390‐095; and **^e^** Institute of Systematic Botany, The New York Botanical Garden, Bronx, NY 10458.

**Table S2.** Taxonomic treatments, nomenclatural updates and morphological descriptions for the eight putatively new taxa within the *Protium heptaphyllum* species complex. Morphological details in this table represent a brief description of key traits commonly used to distinguish tree species within *Protium*. Full taxonomic descriptions and detailed updates will be further published in a monograph review (Damasco et al. in prep).

| New taxonomic treatment based on this study | Previous taxonomic treatment | Previous status | Previous status source | Treatment update | Key vegetative traits | Key reproductive traits |
| --- | --- | --- | --- | --- | --- | --- |
| *Protium cordatum* | *P. heptaphyllum* subsp. *cordatum* | Valid | Daly (1992) | Reestablished (Damasco et al. 2019) | Shrub **habit**, pinnate **leaves** with 1-2 jugas, **petiole** length mean 3.5 cm, **leaflet** coriaceous, elliptical to ovate, **blades** ca. 5-9.5 cm long and 1.5-5 cm wide, **apex** apiculate to cuspidate and **base** cordate. | **Inflorescence** sparse and rare flowers, **pedicel** length mean 0.5-1.2 mm, **corolla** petals with 1.3-2.5 mm, coriaceous with abundant indument, margin barely papillate **anthers** obovate, **fruits** round to obtuse. |
| *Protium* “reticuliflorum” | sp. nov. | - | - | To be published | Understory tree **habit**, pinnate **leaves** with 3-4 jugas, **petiole** length mean 5.2 cm **leaflet** chartaceous, elliptical to oblong, **blades** ca. 8-15 cm long and 3.5-8 cm wide, **apex** caudate and **base** rounded to oblique. | **Inflorescence** dense with abundant flowers, **pedicel** reticulate with length mean 2-4.1 mm, **corolla** petals with 1.2-2.3 mm, chartaceous, glabrous, margin papillate, **anthers** obovate to ovate, **fruits** no info. |
| *Protium* “loretum” | *P. heptaphyllum* subsp. *ulei* | Valid | Daly (1997) | To be published | Stunted to understory tree **habit**, pinnate **leaves** with 1-3 jugas, **petiole** length mean 4.8 cm, **leaflet** chartaceous, lanceolate, **blades** ca. 3.9-9.2 cm long and 2-4.5 cm wide, **apex** abruptly acuminate to caudate and **base** mostly cuneate. | **Inflorescence** sparse and rare flowers, **pedicel** length mean 0.4-1.8 mm, **corolla** petals with 1.2-2.6 mm, chartaceous with slight indument as bristle hairs, margin slightly papillate, **anthers** obovate to ovate, **fruits** round to obtuse. |
| *Protium* “ulei” | *P. heptaphyllum* subsp. *ulei* | Valid | Daly (1997) | To be reinstated | Stunted to understory tree **habit**, pinnate **leaves** with 1-3 jugas, **petiole** length mean 4.4 cm, **leaflet** mostly coriaceous and barely chartaceous, elliptical to ovate, **blades** ca. 4-12 cm long and 1.9-4.8 cm wide, **apex** broadly acuminate and **base** rounded. | **Inflorescence** sparse and rare flowers, **pedicel** length mean 0.5-2.1 mm, **corolla** petals with 1.5-2.9 mm, chartaceous with slight indument, margin mostly papillate towards the apex, **anthers** obovate to ovate, **fruits** round and barely obtuse. |
| *P. angustifolium* | *P. angustifolium* | Synonym | Swart (1942) | To be reestablished | Understory tree **habit**, pinnate **leaves** with 3-4 jugas, **petiole** length mean 4.8 cm, **leaflet** chartaceous, lanceolate, **blades** ca. 7.4-12.3 cm long and 2.1-3.4 cm wide, **apex** narrowly acute and **base** cuneate. | No flower information available. |
| *P.* “tucuruiense” | sp. nov. | - | - | To be published | Shrub **habit**, pinnate **leaves** with 1-3 jugas, **petiole** length mean 3.1 cm, **leaflet** coriaceous, elliptical to ovate, **blades** ca. 5.2-9.6 cm long and 2-4.5 cm wide, **apex** apiculate to cuspidate and **base** rounded to broadly acute. | **Inflorescence** sparse and rare flowers, **pedicel** length mean 0.4-1.5 mm, **corolla** petals with 1-2.2 mm, coriaceous and glabrous, margin papillate at the apex, **anthers** obovate, **fruits** round to obtuse. |
| *Protium heptaphyllum* | *P. heptaphyllum* subsp. *heptaphyllum* | Valid | Daly (1992) | No update | Understory to canopy tree **habit**, pinnate **leaves** with 3-4 jugas, **petiole** length mean 5.2 cm, **leaflet** chartaceous, broadly elliptical to oblong, **blade** ca. 7.1-12.3 cm long and 3.4-5.7 cm wide, **apex** broadly acute to apiculate and **base** rounded and commonly asymmetric. | **Inflorescence** dense with abundant flowers, **pedicel** length mean 2-7.1 mm, **corolla** petals with 2.5-3.9 mm, chartaceous, glabrous with dark venations, margin densely papillate, **anthers** sharply lanceolate, **fruits** red to purple when mature with obtuse apex. |
| *Protium* “aromaticum” subsp. “floribundum” | *P. heptaphyllum* var. *floribundum* | Synonym | Swart (1942) | To be published | Canopy tree **habit**, pinnate **leaves** with 3-5 jugas, **petiole** length mean 6.8 cm, **leaflet** chartaceous, oblong to elliptical, **blade** 7.1-16.4 cm long and 3.9-6.5 cm wide, **apex** cuspidate to slightly caudate and **base** rounded. | **Inflorescence** dense with very abundant flowers, **pedicel** length mean 2-4.1 mm, **corolla** purple petals with 2.5-3.8 mm, chartaceous, glabrous, margin papillate, **anthers** sharply oblong, **fruits** purple to red when mature and obtuse apex. |
| *Protium* “aromaticum” subsp. “aromaticum” | *P. heptaphyllum* var. *aromaticum* | Synonym | Swart (1942) | To be published | Stunted to canopy tree **habit**, pinnate **leaves** with 2-5 jugas, **petiole** length mean 4 cm, **leaflet** chartaceous to coriaceous, mostly elliptical, **blade** ca. 4.3-10.1 cm long and 2.3-5.1 cm wide, **apex** broadly acute to cuspidate and **base** rounded to acute. | **Inflorescence** dense with abundant flowers, **pedicel** length mean 1.9-3.8 mm, **corolla** white to greenish petals with 2.7-3.9 mm, chartaceous, sparse indument, margin papillate, **anthers** sharply oblong, **fruits** red and commonly round with occasional obtuse apex. |
