# Supplementary material for: Revisiting the hyperdominance of Neotropical tree species under a taxonomic, functional and evolutionary perspective
Source: Sci Rep. 2021 May 5;11:9585. doi: 10.1038/s41598-021-88417-y (PMC8099866; doi:10.1038/s41598-021-88417-y)
Supplement: Supplementary file 6 — Supplementary Table S4. [file 41598_2021_88417_MOESM6_ESM.docx]

**Revisiting the hyperdominance of Neotropical tree species under a taxonomic, functional and evolutionary perspective**

**Gabriel Damasco^a,b^
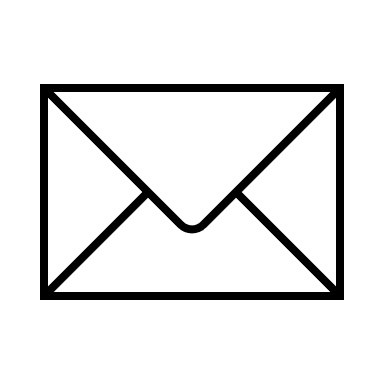
, Christopher Baraloto^c^, Alberto Vicentini^d^, Douglas C. Daly^e^, Bruce G. Baldwin^a^, Paul V. A. Fine^a^**

**^a^** Department of Integrative Biology, University of California, Berkeley, CA 94720-3140, email: [gdamasco@berkeley.edu](mailto:gdamasco@berkeley.edu), [gabrielfloresta@gmail.com](mailto:gabrielfloresta@gmail.com); **^b^** Department of Biology and Environmental Science, University of Gothenburg, St. Paul, MN 55108; **^c^** International Center of Tropical Biology, Florida International University, Miami, FL 33133; **^d^** Instituto Nacional de Pesquisas da Amazônia, Programa de Pós–graduação em Ciências Biológicas (Botânica), Manaus, AM 70390‐095; and **^e^** Institute of Systematic Botany, The New York Botanical Garden, Bronx, NY 10458.

**Table S4. Functional trait description for different populations of *Protium heptaphyllum s.l*.** Leaf and wood functional traits are displayed bellow. Information regarding the number of replicates, trait variation range, and proxy for trait strategies is described in detail.

| Functional trait | Unit | Group | Replicates per population | Trait range  (min - max) | Strategy |
| --- | --- | --- | --- | --- | --- |
| Specific Leaf Area (SLA) | cm^2^g^-1^ | Leaf | 12 | 70.78 – 148.88 | Investment in photosynthetic capacity versus resource conservation and herbivory defense. |
| Stable Isotope Composition (δ^13^C) | ‰ | Leaf | 3 | -36.33 – -28.88 | Intrinsic water use efficiency (WUE) and water-related limitations. |
| Leaf Nitrogen Content (N) | % | Leaf | 3 | 1.21 – 1.88 | Photosynthetic capacity. |
| Chlorophyll Content Index (CCI) | % | Leaf | 12 | 19.19 – 29.58 | Photosynthetic capacity. |
| Stomatal Density (SD) | mm^-2^ | Leaf | 5 | 140.2 – 263.5 | Assuming similar values of stomatal size, indicates higher CO_2_ uptake. |
| Vessel diameter | µm | Wood | 5 | 26.87 – 38.63 | Hydraulic efficiency. |
| Vessel length | µm | Wood | 5 | 71.17 – 131.42 | Hydraulic efficiency and vulnerability to cavitation. |
| Vessel density | mm^-2^ | Wood | 5 | 179 – 264.7 | Hydraulic efficiency. |
